# *Capra* cartilage-derived peptide delivery via carbon nano-dots for cartilage regeneration

**DOI:** 10.3389/fbioe.2023.1213932

**Published:** 2023-08-28

**Authors:** Priti Prasanna Maity, Kausik Kapat, Puja Poddar, Hema Bora, Chandan Kanta Das, Poushali Das, Sayan Ganguly, Narayan Chandra Das, Dibakar Dhara, Mahitosh Mandal, Amit Roy Chowdhury, Sumanta Mukherjee, Santanu Dhara

**Affiliations:** ^1^ School of Medical Science and Technology, IIT Kharagpur, Kharagpur, India; ^2^ Department of Medical Devices, NIPER Kolkata, Kolkata, India; ^3^ Department of Chemistry, IIT Kharagpur, Kharagpur, India; ^4^ Rubber Technology Centre, IIT Kharagpur, Kharagpur, India; ^5^ Aerospace Engineering and Applied Mechanics, IIEST Shibpur, Howrah, West Bengal, India; ^6^ Production Engineering Department, BIT Sindri, Dhanbad, Jharkhand, India

**Keywords:** carbon nano dots, zwitterion, peptide synthesis, collagen II, cartilage regeneration

## Abstract

Targeted delivery of site-specific therapeutic agents is an effective strategy for osteoarthritis treatment. The lack of blood vessels in cartilage makes it difficult to deliver therapeutic agents like peptides to the defect area. Therefore, nucleus-targeting zwitterionic carbon nano-dots (CDs) have immense potential as a delivery vehicle for effective peptide delivery to the cytoplasm as well as nucleus. In the present study, nucleus-targeting zwitterionic CDs have been synthesized as delivery vehicle for peptides while also working as nano-agents towards optical monitoring of cartilage healing. The functional groups of zwitterion CDs were introduced by a single-step microwave assisted oxidation procedure followed by COL II peptide conjugation derived from *Capra* auricular cartilage through NHS/EDC coupling. The peptide-conjugated CDs (PCDs) allows cytoplasmic uptake within a short period of time (∼30 m) followed by translocation to nucleus after ∼24 h. Moreover, multicolor fluorescence of PCDs improves (blue, green, and read channel) its sensitivity as an optical code providing a compelling solution towards enhanced non-invasive tracking system with multifunctional properties. The PCDs-based delivery system developed in this study has exhibited superior ability to induce *ex-vivo* chondrogenic differentiation of ADMSCs as compared to bare CDs. For assessment of cartilage regeneration potential, pluronic F-127 based PCDs hydrogel was injected to rabbit auricular cartilage defects and potential healing was observed after 60 days. Therefore, the results confirm that PCDs could be an ideal alternate for multimodal therapeutic agents.

## 1 Introduction

Cartilage repair after degeneration or trauma continues to be a challenge in both clinic and scientific research due to limited regenerative capacity of the tissue. Considering the limitations in tissue regeneration process, various tissue-engineered products are being used in clinics to treat the damaged cartilage. Peptide-based treatment approach have gained a strong attention in the recent times as the biomolecules can be designed to trigger specific cell-signaling pathways leading to chondrogenesis. However, therapeutic potential of peptides have not yet been fully realized owing to their poor cell membrane permeability that limits their intracellular exposure (Sun et al., 2022). Therefore, it is commonly used as an oral dietary supplement for treatment of various diseases. However, the therapeutic efficiency of the oral delivery is limited by the degradation the agents in gastrointestinal tract as well as their inability to cross the epithelial barrier (Bruno, Miller and Lim, 2013). Molecular structure-wise, peptides have short chain amino acid sequence, can completely mimic the protein functionality and modulate the cellular behavior (Liu et al., 2018; Okoye et al., 2022). A number of synthetic peptides have also been engineered in the form of scaffold as well as functional molecules to treat cartilage defects (Liu et al., 2018; Xue et al., 2021). However, the major hurdle in the peptide-based therapeutics still remains in the form of inefficient delivery to the targeted tissue. Except synthetic cell penetrating peptides, natural peptides isolated from proteins have limited capacity to penetrate to the tissue. Another significant challenge in delivering the biomolecules including peptides to the cartilage defects is the lack of blood vessels in the tissue (Jones and Winzenberg, 2019). Therefore, an urgent and unmet need is there to develop delivery vehicle to deliver biomolecular therapeutic agents including peptides to cartilage defects.

Carbon nanodots (CDs) can be a promising tissue non-invasive delivery vehicle for such peptides and other biomolecules. Apart from this delivery potential, CDs possesses high fluorescence-stability, non-cytotoxicity, and biocompatibility, and thus, can be an efficient and cost-effective alternative to the conventional synthetic florescent dye to label and monitor the live cells through cytoplasmic localization (Baker and Baker, 2010). For nuclear localization and effective small molecules delivery, surface-functionalized zwitterion CDs has certain advantages in terms of internalization into nucleus through receptor mediated endocytosis in non-aggregated form (Jung, Shin and Kim, 2015). Zwitterions are molecules with two predicted pKa values, one acidic and other basic, leading to a net neutral (zero) charge on the molecule (Shao et al., 2014). In water medium, zwitterions provide strong hydration through electrostatic interaction producing physical and energetic barrier against protein adsorption. Therefore, zwitterionic CDs has less ability to adsorb proteins on their surface, promoting faster agglomeration-free uptake in cell nuclei (Sri et al., 2018). Because of the multiple functional groups present on the surface, it is often used in pharmaceutical, therapeutics and diagnostics agents delivery for various treatment modalities (Banerjee, Ravishankar Rai and Umashankar, 2019). As a significant surface area of the CDs is covered with carboxylic groups, molecules bearing amino groups can passivate onto carbon nanodots through amide linkage (Wu et al., 2016). Carboxylic functionalities on CDs surface also expands the palette of bio-conjugating biomolecules including drugs (Sangtani et al., 2018), genetic materials (Kailasa et al., 2019), enzymes (Vaughan, 2021), antibodies, peptides (Kuo et al., 2011; Agarwal et al., 2015; Jeong et al., 2018; à et al., 2019; Khan et al., 2022) etc.

This work presents synthesis of surface-functionalized zwitterions CDs and conjugation with collagen II peptides to evaluate its bifunctionalities in cartilage regeneration. Further, the capacity of differentiation of Adipose-derived mesenchymal stem cells (ADMSCs) to chondrogenic lineages by addition of peptide-conjugated CDs (PCDs) is reported for the first time. The results show promising aspects for therapeutic application of site-specific delivery of peptides in cartilage tissue engineering field.

## 2 Materials and methods

### 2.1 Synthesis of zwitterion CDs from *Capra* auricular cartilage

Auricular cartilage tissue was obtained from freshly collected *Capra* ears by eliminating the skin with hypertonic solution, and the tissue was physically pulverized, decellularized with NaOH, and lyophilized as described in our previous works (Maity et al., 2019; Maity et al., 2022). For CDs synthesis, different concentrations (25, 50, 100, 150 and 200 µL per 20 mL of water) of HNO_3_ were added to 300 mg of the lyophilized mass, and sonicated in probe sonicator (Oscar Ultrasonics, India) for 1 h followed by microwave irradiation (750 W) for 6 min. Next, the partially carbonized fractions were ground, mixed with 20 ml deionized water (diH_2_O), sonicated for 1 h and pH was adjusted to 7.4. Finally, obtained CDs was dialyzed using dialysis membrane of 3.5 kDa molecular cut-off against diH_2_O followed by lyophilization.

### 2.2 Collagen II peptide isolation and conjugation with zwitterion CDs

**FIGURE 1:**
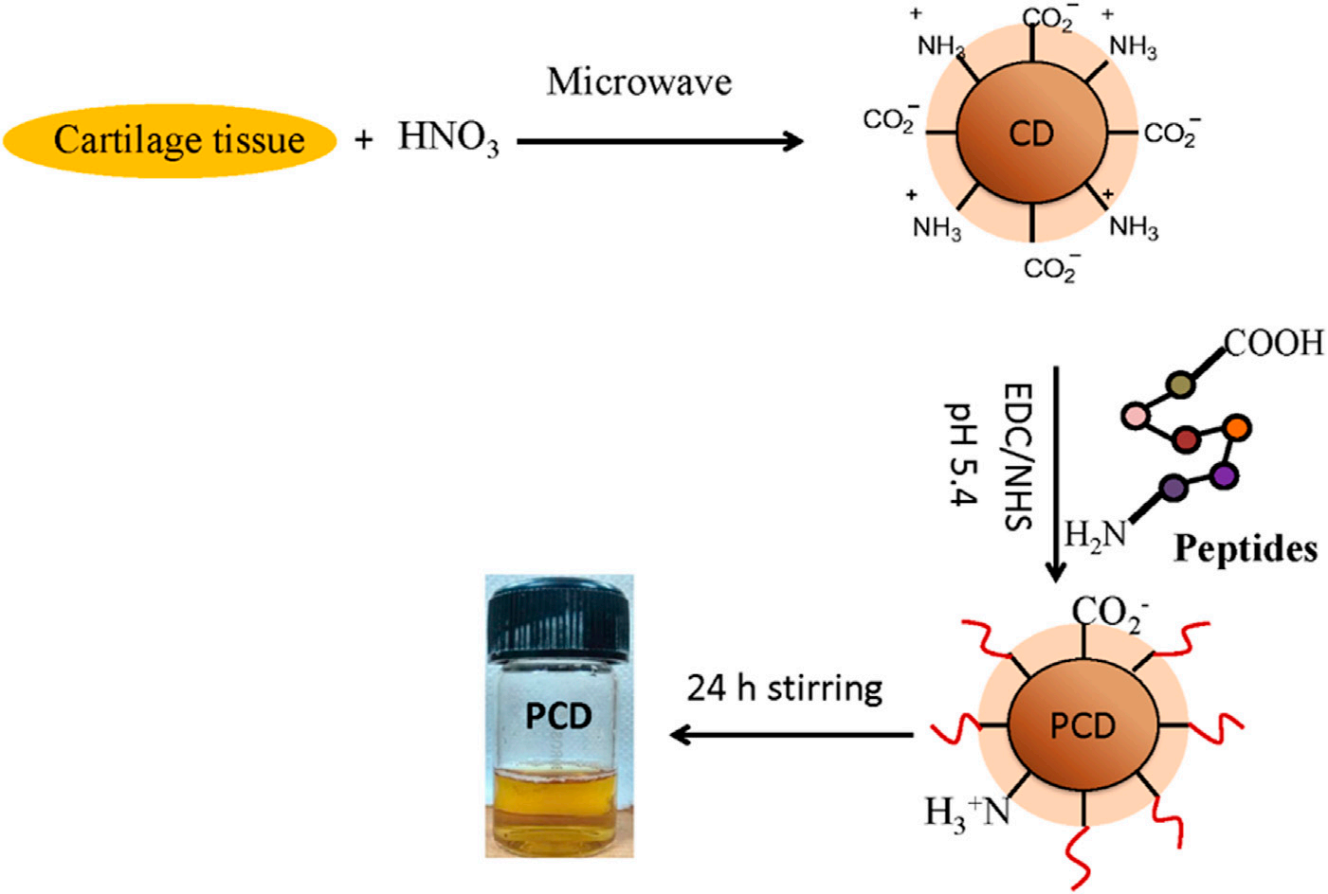
Workflow of MAP-based peptide isolation and PCD synthesis: *Capra* cartilage tissue was mixed with HNO_3_ and microwave irradiated to generate zwitterion CDs. COL II peptides conjugate on the surface of CDs by NHS/EDC coupling followed by continuous stirring of the micelles to form peptide conjugated CD (PCD).

*Capra* auricular cartilage-derived Collagen II (COLII) was used to synthesis by microwave-assisted acid pyrolysis (MAP) (Chen et al., 2014) and were conjugated with CDs (Figure 1). Briefly, the lyophilized COLII was vortexed with 25% Trifluoroacetic acid (TFA) + 20 mM Dithiothreitol (DTT) for 5 m to obtain a homogeneous solution. The solution was transferred to 1.5 mL polypropylene vials and sealed with Teflon tape for processing using a domestic microwave oven (900 W, 2,450 MHz) for 3, 5, 7, and 10 m. A beaker containing 100 mL water was also placed in microwave oven during the irradiation. After microwave processing, the solutions were dried in SpeedVac for 30 m for acid removal, dissolved with 0.1% TFA, desalted in Amicon filter (cut off. 3 kDa, Merck, USA), and dried again in the SpeedVac.

After spotting of the dried samples on to the Anchor chip target plate (600/384 F, Bruker Daltonics), the spots were dried for 30 m and analyzed for MALDI-TOF by using Ultraflextreme mass spectrometer (Bruker Daltonics, Germany). Different areas of sample spot on MALDI target plate were selected for data acquisition.

The isolated peptides were conjugated with CDs by NHS/EDC coupling method. Briefly, CDs (1 mg) was diluted with 1 ml diH_2_O and pH was adjusted to 5.4. For synthesis of PCDs by time-based MAP followed by NHS/EDC coupling, 1:5:5 ratio of CDs, NHS and EDC was observed to be optimal for efficient conjugation, since EDC with NHS forms an active ester with -COOH groups present on CDs surface (Bartczak and Kanaras, 2011). The NHS/EDC was added to the diluted CDs solution followed by continuous magnetic stirring at 37°C for 30 m. The dried peptides (1 mg) were introduced into the solution and magnetically stirred for 10 h followed by incubation at 4°C to stop the reaction. The peptide-conjugated CDs were dialyzed (Dialysis membrane, 3.5 kDa molecular cut-off) against diH_2_O for 12 h and lyophilized.

### 2.3 Characterization of peptide-conjugated zwitterion CDs (PCDs)

Dynamic light scattering (DLS) and Zeta potential were recorded with a Malvern Zetasizer Nano-equipment with a temperature-controlled sample chamber by employing a 4.0 mW He−Ne laser operated at 632.8 nm and 173° angle. The raw data were processed to obtain the hydrodynamic diameter (D_h_) and size distribution in terms of polydispersity index (PDI). UV-vis absorption spectrum of PCDs was performed on UV-visible spectrophotometer (Shimadzu UV-2450) and the 300–600 nm spectrum was analyzed. Further, Photoluminescence (PL) spectra were measured on a Hitachi F-4600 fluorometer equipped with Xe lamp at ambient conditions. Transmission electron microscopy (TEM, FEI Tecnai G2 F30) was performed for morphological analysis of PCDs. Attenuated Total Reflection Fourier Transform Infrared Spectroscopy (ATR-FTIR) of PCDs was performed over 4,000–700 cm^-1^ range using Spectrophotometer (Thermo Nicolet NEXUS-870). Finally, nano-morphology of PCDs was analyzed by atomic force microscopy (AFM, Bruker Dimension Icon).

### 2.4 Assessment of cytotoxicity and cyto-compatibility of PCDs

*Capra* adipose tissue-derived mesenchymal stem cells (ADMSCs) were used for the assessment of cytotoxicity and cyto-compatibility of PCDs at 1, 3, and 5 d post-incubation. Necessary approval for the isolation of ADMSCs was obtained from the Committee for Stem Cell Research (CSCR) of Indian Institute of Engineering Science and Technology (Shibpur), India vide approval no. CSCR/Decisions/3/15. The isolation and characterization of ADMSCs from *Capra* have been detailed in our previous work (Maity et al., 2019). Cytotoxicity of PCDs was assessed by MTT assay as per the protocol (Maity et al., 2022). Briefly, freshly prepared MTT solution (40 μL, 5 mg/mL) was added to the cells and incubated for 4 h. After carefully removing the supernatant, the formazan crystals were dissolved with DMSO and absorbance was measured using microplate reader (Bio-Rad 680, USA) at 570 nm. Live-dead assay (Invitrogen) was performed to assess viability of ADMSCs in presence of PCDs according to the protocol (Roy et al., 2019). Briefly, 1, 3 and 5 d post-incubation, PCDs and tissue culture plate (TCP) as control were washed with PBS followed by incubation with live–dead staining solution at 37°C for 30 m. After incubation, the cells were washed with PBS and observed under fluorescent microscope (Carl Zeiss) with excitation filters of 450–490 nm (green, Calcine AM) and 510–560 nm (red, ETD-1). Live-dead assay was also performed with MG63 cells for both CD and PCD-containing media after 1 and 5 days culture.

### 2.5 PCDs uptake study

For internalization of PCDs in cells cytoplasm and nucleus, ADMSCs (1 × 10^4^) were seeded into each well of six-well plates and incubated for 24 h at 37 °C. After removal of culture media, the plates were washed with PBS followed by addition of 20 μL of PCDs (100 μg/mL) to each well at 3, 6, 12, 24, and 48 h. After incubation, the cells were visualized under confocal microscope (TCS SP8, Leica).

### 2.6 PCDs-induced spheroid formation and *ex-vivo* chondrogenic differentiation study

After trypsinization, ADMSCs were plated into six-well plates. At 70% confluence, DMEM media containing PCDs was added to the cells and incubated at 37°C in CO_2_ incubator for generation of cell clusters. The clusters were transferred to pluronic F 127 hydrogel for long time culture in 3D environment for spheroid formation. For this purpose, lyophilized PCD (0.1 mg/ml) was mixed with F 127 (20% w/v) containing DMEM media to form pre-gel mix. The blend was mixed vigorously and transferred to a refrigerator for micelles formation. After complete dissolution, the solution was layered in 24 well plate followed by 1 h incubation at 37°C in CO_2_ incubator for complete gelation. Cell clusters were placed on the surface of hydrogel and incubated at 37°C in CO_2_ incubator for 30 m. Pre-warmed DMEM media containing PCDs was added to the system for 21 d study, while the PCDs/media was replaced every 3rd day. RNA isolation, CDNA synthesis and qPCR of cartilage specific gene amplification with GAPDH normalization was performed as per our previously published literature (Kapat et al., 2018; Mukherjee, Dhara and Saha, 2021) with the primers mentioned in appended Supplementary Table SA1.

Quantitative analysis of sulfated glycosaminoglycan (sGAG) was carried out by Alcian blue assay for evaluating the extent of chondrogenesis induced by the PCDs. For this assay, the spheroids were digested for 12 h with cysteine hydrochloride (10 mM, Sigma, USA), papain (125 μg/ml, Sigma, USA) and Na_2_EDTA (2 mM, Sigma, USA) in a phosphate buffer solution (0.1 M, pH 6.8) at 60°C for 12 h. The digested solution was centrifuged at 15,000 g for 20 min to collect the supernatant. A 10% solution of Alcian Blue dye stock (1 g Alcian Blue 8GS dye in 100 ml of 18 mM H_2_SO_4_) was in 0.25% Triton X-100, 0.018 M H_2_SO_4_ was prepared, and guanidine solutions (8 M in 0.027 M H_2_SO_4_ and 0.375% Triton X-100) were also prepared. The Alcian blue solution was mixed with the previously collected supernatant and vortexed for 5 m followed by centrifuging at 16,000 g for 10 m at 4°C. The obtained pellets were dissolved in 500 µl of 8 M guanidine HCl by vigorous vortexing followed by centrifugation at 16,000 g for 3 m. Absorbance of the Alcian blue-sGAGs complex was measured at 595 nm wavelength using microplate reader (iMarkt, India) and the quantities of sGAGs contents were estimated by comparing the obtained data with a standard curve.

### 2.7 Regeneration of auricular cartilage defects in rabbit

New Zealand white rabbits (n = 4) of age 12 months and weighing 2.5–3 kg were procured from Chakraborty Enterprise (Kolkata, India) and were used for the animal study. Necessary animal ethical clearance was obtained from Institutional Ethical Committee of Indian Institute Technology, Kharagpur (IIT Kharagpur), India (IE-4/SD-SMST/2.15). The animals were acclimatized for 2 weeks in the animal care facility with adequate diet prior to the surgery. For the surgical procedure, acclimatized rabbits were anesthetized by Midazolam (0.5 ml) followed by local anesthesia (Lignocaine) of the periphery of surgical zone. After sterilization by β-Iodine, cartilage defects (8 mm) were made near the center of each auricle without damaging the outer skin using a drilling machine with a drill bit of 8 mm dia. Details of the surgical procedure can be found one of our previous works (Maity et al., 2022). PCD-F127 hydrogels were injected into the defects in both right and left ears (n = 8 ears) and subsequently covered with leucoplast over a thin layer of cotton gauge. For post-operative pain management, the rabbits were treated with Ibuprofen (1ml/day) and Calmpose (0.25ml/day) for 3 days under the guidance of a veterinary surgeon.

To evaluate cartilage regeneration, the rabbits were euthanized after 15 and 60 d post-surgery with an overdose of Midazolam injection and the tissue specimens were collected and fixed in 4% v/v formalin. Fixed tissue specimens were dehydrated by treating with graded series of ethanol (70–100%) followed by tissue cleaning with xylene and finally embedded in paraffin for histological sectioning. The obtained sections from paraffin blocks were stained with hematoxylin and eosin (H&E) and Masson’s trichrome (MT, Sigma-Aldrich, USA) to assess cartilage regeneration. The stained histological samples were visualized and captured for images using an Axio Observer Z1 microscope (Carl Zeiss, Germany).

### 2.8 Statistical analysis

All the experiments were performed in triplicate. The results are reported as mean ± standard deviation (SD) and statistical analysis was carried out by performing one-way ANOVA using Origin 9.1 software (USA). *p* < 0.05 was considered statistically significant.

## 3 Results and discussion

### 3.1 Synthesis and characterization of zwitterion CDs

**FIGURE 2:**
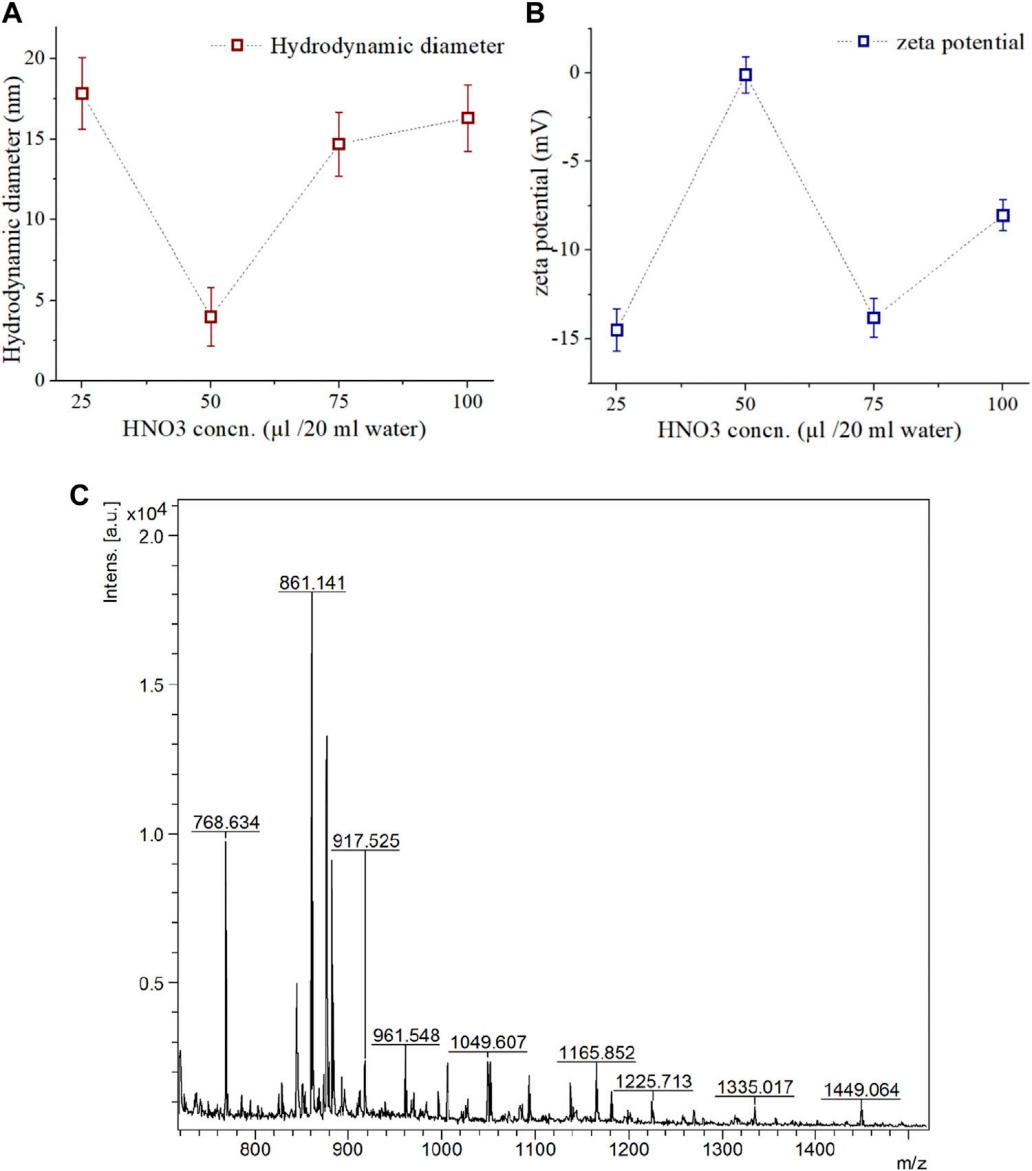
Characterization of synthesized CDs and isolated COLII peptides. **(A)** Hydrodynamic diameter, and **(B)** Zeta potential of synthesized CD assessed at different concentration of HNO3 treatment. **(C)** MAP based isolated COLII peptides identified by MALDI-TOF analysis.

It was observed that both the hydrodynamic diameter (Figure 2A) and zeta potential (Figure 2B) of synthesized CDs depends upon the concentration of HNO_3_. The optimum concentration of HNO_3_ was found to be 50 µg/20 mL water for synthesizing zwitterion CDs with 3.98 nm average diameter and zeta potential of −0.119 mV. At low acid concentration of 25 µg/20 mL water the average diameter was higher and the zeta potential was lower, while both of these features displayed gradually increasing trends at higher concentrations.

It can be assumed that low concentration of HNO_3_ resulted in inefficient pyrolysis, evidenced by larger hydrodynamic diameters and lower zeta potential. On the other hand, high acid concentration can induce negatively charged -OH, -COOH, -NO_2_ functional groups on the surface of the CDs (Ray et al., 2009).

### 3.2 Peptide isolation from COLII of *Capra* auricular cartilage

In microwave-assisted acid pyrolysis (MAP) for peptide isolation, the yield was found to be highest for 5 min of irradiation (Figure 2C), and longer exposure caused disintegration as evident from the MALDI-TOF MS analysis. Such observations are consistent with existing literature (Reiz and Li, 2010).

### 3.3 Conjugation and characterization of PCDs

**FIGURE 3:**
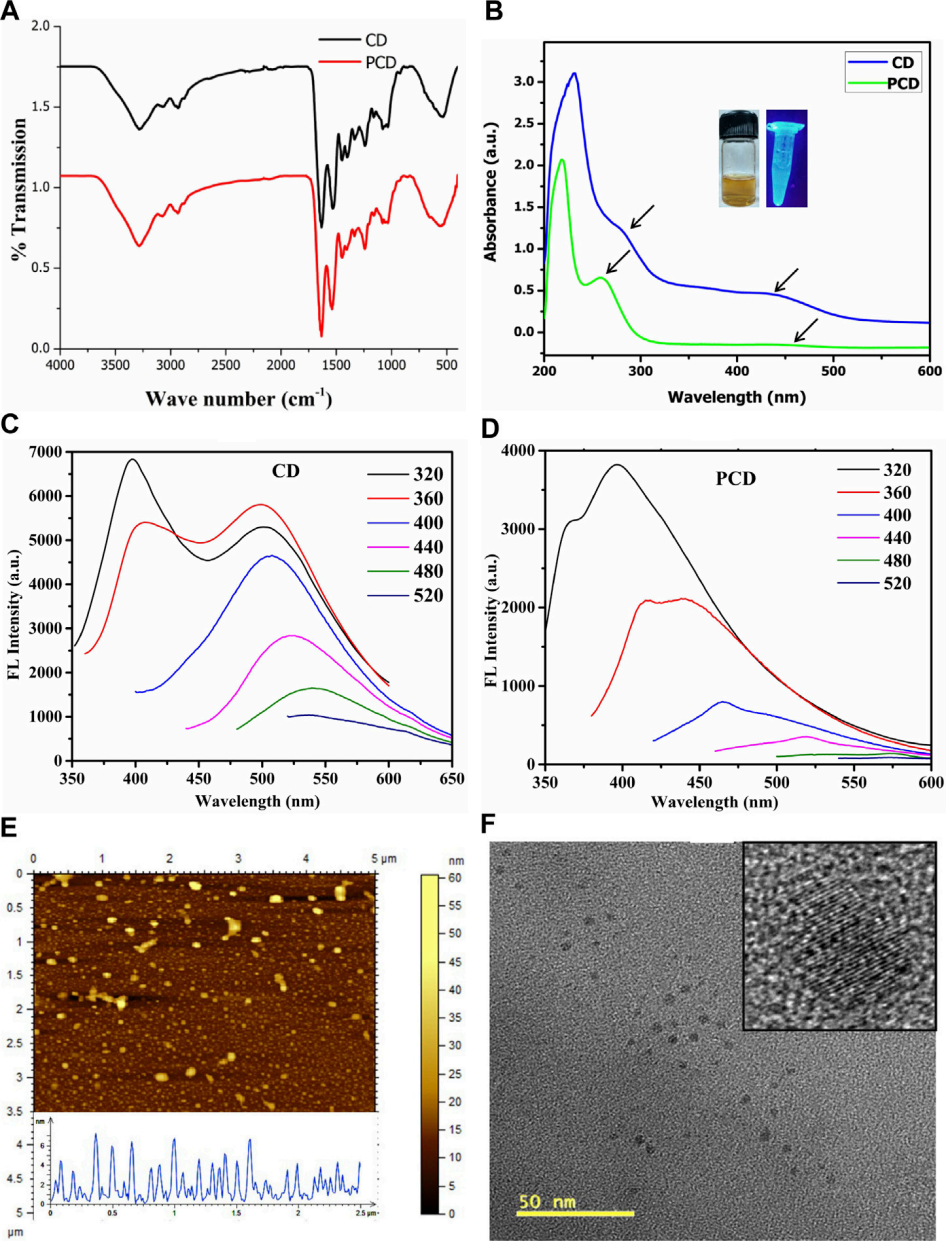
Physico-chemical characterization of CD and PCD. **(A)** FTIR Spectroscopy, **(B)** UV absorption spectra, **(C,D)** Photoluminescence emission spectra, **(E)** Particles size distribution of PCD by AFM and **(F)** TEM images of the PCD.

Peptide conjugation on the surface of CDs by NHS/EDC coupling for 24 h was optimum to generate sufficient yield of PCD. To identify the structural and functional characteristics in PCDs, several non-destructive testing like FTIR, UV, PL, AFM, and TEM were performed. Hydroxyl and other polar functional groups (-C=O, -COOH, -C-O-C-) generated through the single step top-down oxidative coupling on PCDs surface were detected through FTIR analysis (Figure 3A). FTIR spectra showed that the combined pyrolysis and carbonization process introduced oxygen-containing functional groups at 1,010–1,106 cm^-1^ for C–O–C, 1,240 cm^-1^ for C–OH and 1720 cm^-1^ for C=O, respectively, to the edges and basal plane of PCDs. The characteristic peaks at ∼3,280 cm^−1^ and 1,535–1,640 cm^-1^ were assigned to -OH and amide functionalities, respectively. Other peaks at 810, 1,012, and 1,364 cm^-1^ correspond to the presence of C-H bending and C-O stretching frequencies arising from the aromatic amino acids. These polar groups contributed to an increased water solubility of the synthesized PCDs. Further, the peaks at 1,610–1,650 cm^-1^ indicate the presence of sp^2^ hybridized honeycomb lattice (Strauss et al. 2014).

The influence of surface polarity over optical properties of PCDs was assessed by UV-vis spectroscopy, as shown in Figure 3B The absorption peaks at 258 and 460 nm with a tail extending to visible range were attributed to π–π* transition of -C=C bonds and n–π* transition of C=N bonds, respectively. The peak at 258 nm was related to the absorption of aromatic system with sp^2^ carbon network and peak at 460 nm was attributed to carbonyl group present on the PCDs surface (Jung, Shin and Kim, 2015). It was evident from the fluorescence spectra that the PCDs showed different excitation wavelength (Figures 3C, D). The excitation wavelength of PCDs varied from 380 to 640 nm and emission spectra shifted to longer wavelengths. The maximum emission peak was found at 620 nm for excitation at 520 nm.

The TEM and AFM images in Figures 3E, F shows quasi-spherical dot-like morphology of PCDs in the size range 2–8 nm. The size distribution obtained through DLS study was calculated in the range of 2.5–8.0 nm, which is little bit higher than the data obtained from TEM analysis. Due to high surface polarity of the PCDs, they have easy solvation in protic media.

### 3.4 Assessment of cytotoxicity and cyto-compatibility of CDs and PCDs

**FIGURE 4:**
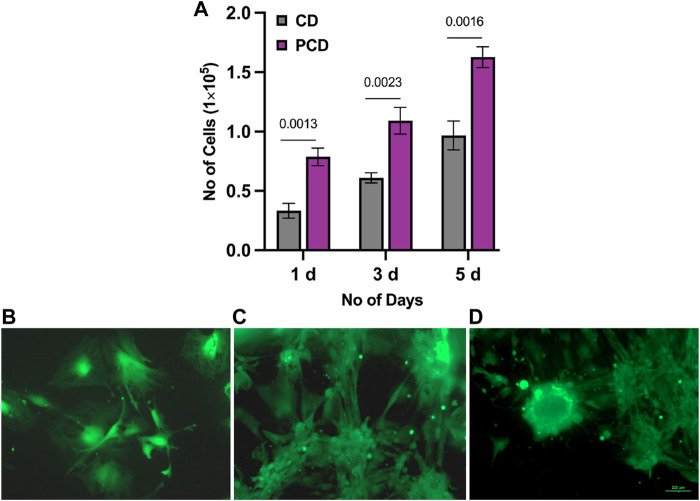
Assessment of cytotoxicity and cyto-compatibility of PCD. **(A)** MTT assay results for CD and PCD at 1, 3, and 5 d, horizontal line indicating the *p*-value between two groups, **(B–D)** live-dead assay of PCD at 1, 3, and 5 d of culture.

MTT assay (Figure 4A) established that the cell populations with PCDs at 1, 3, and 5 d were significantly higher than those with CDs (*p* ≤ 0.05). Cell population almost doubled on day 5 from day 3. Live-dead assay (Figures 4B–D) also showed relatively higher proliferation of cells with cluster formation on 5^th^ day as compared to 3 d of culture. These results confirmed the cytocompatibility of PCDs, and indicated that the PCDs could be internalized by cell nuclei and could modulate the cell behavior to form clusters. Results of the Live-dead assay for MG63 cells are appended as Supplementary Figure SA1.

### 3.5 PCDs uptake study using ADMSCs

**FIGURE 5:**
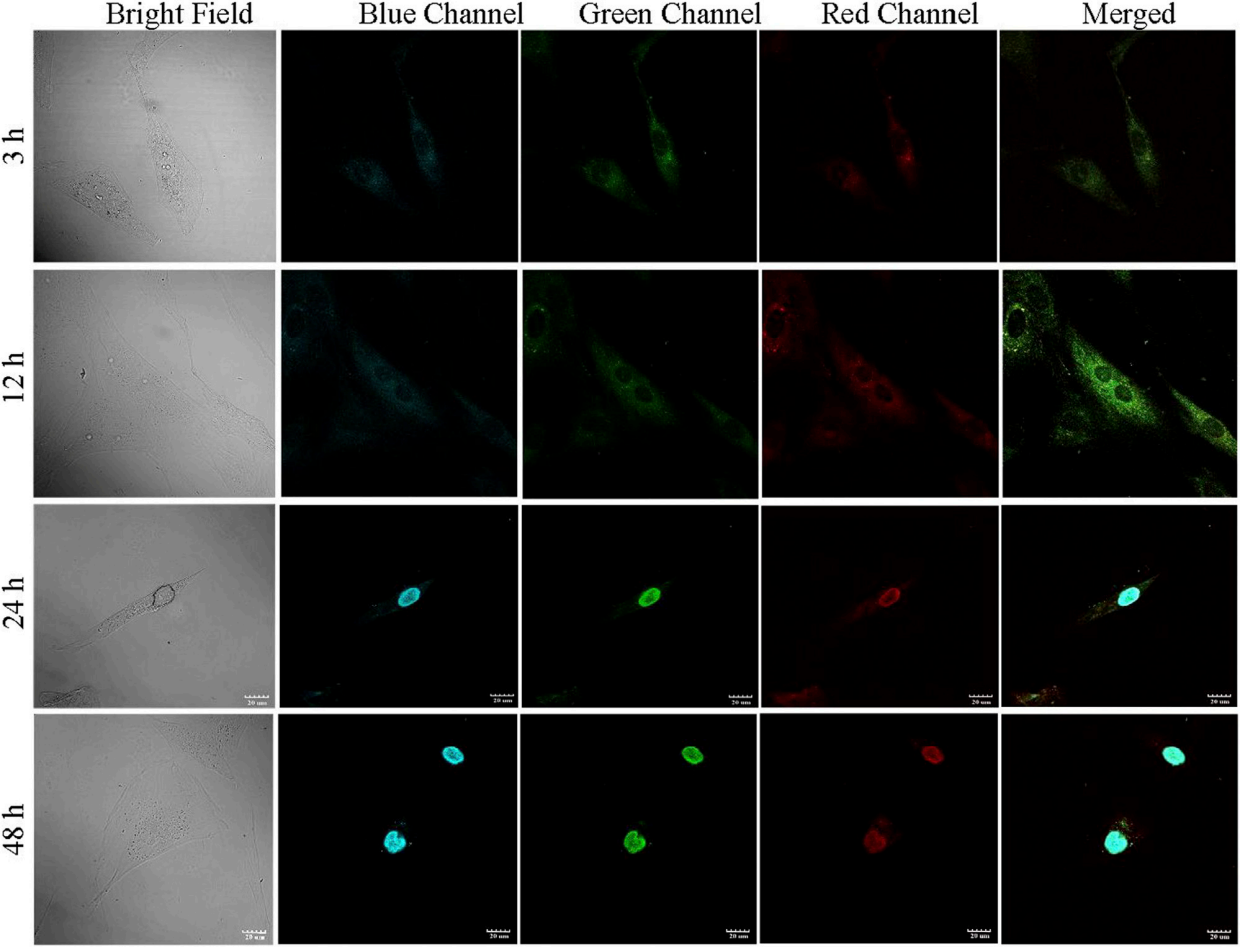
PCD uptake study performed in ADMSCs. The fluorescence of PCD appeared in cytoplasm at 3 h, migrated to nucleus at 24 h, and complete relocation to nucleus observed at 48 h.

During confocal imaging, excitation of PCDs with 365, 488, and 580 nm wavelengths produced blue, green and red fluorescence, respectively. The fluorescence of PCDs started to appear in cell cytoplasm at 30 m and subsequently migrated to nucleus within 24 h of incubation. The highest localization of PCDs in nucleus was obtained at 48 h (Figure 5). Neutrally charged carbon nano-dots with size less than 9 nm is known to be transported to nuclei within 24 h of seeding (Jung, Shin and Kim, 2015), and therefore the multicolor fluorescent PCDs can be effective in non-invasive monitoring of tissue in-growth and healing of wound.

### 3.6 *Ex vivo* spheroid formation and *In vitro* chondrogenic differentiation

**FIGURE 6:**
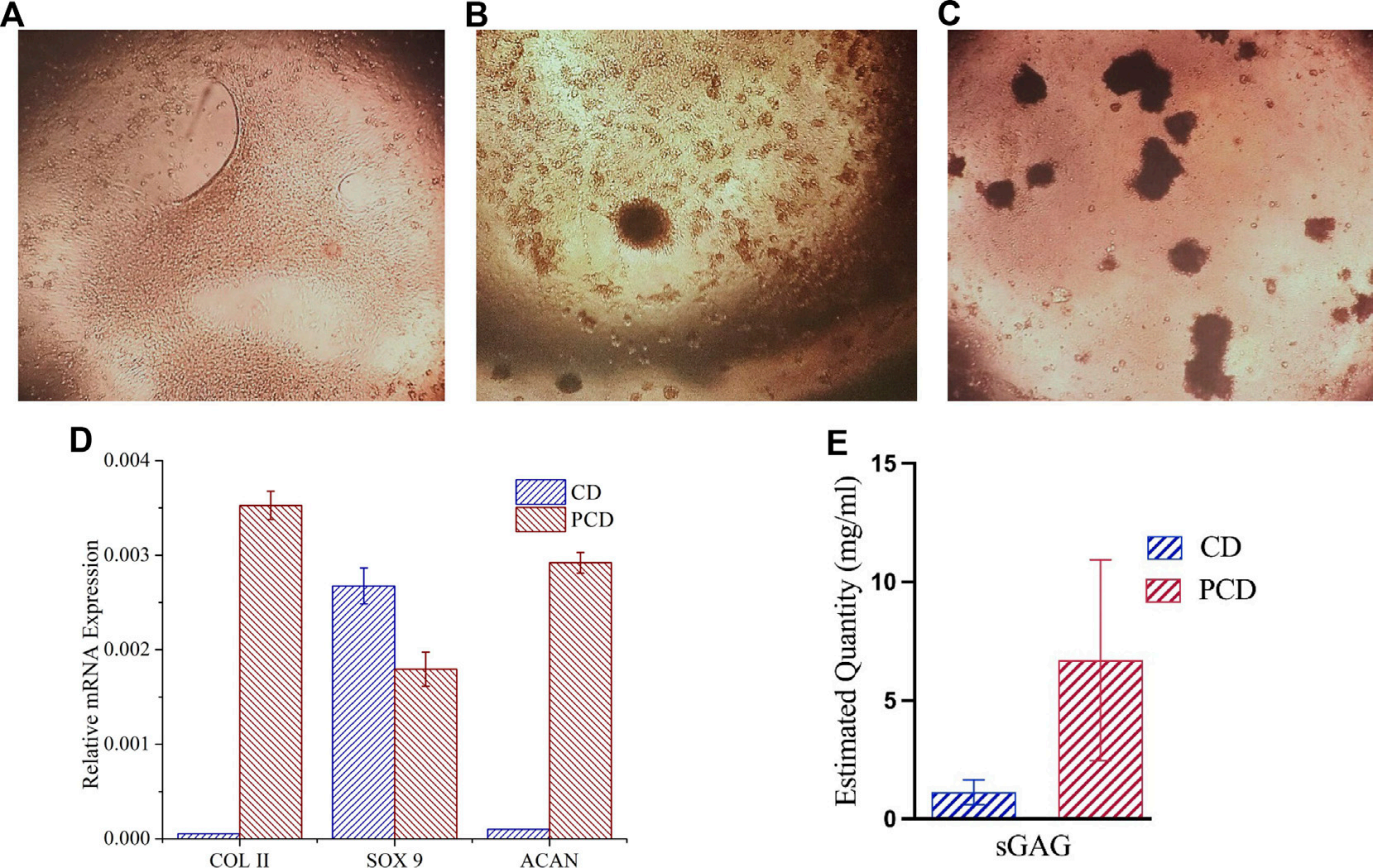
Chondrogenic differentiation of cells and cluster formation in PCD-F127 hydrogel. **(A)** Cell mono layers shrinking towards cluster formation, **(B)** Few clusters formed after 3 d culture and **(C)** Several clusters formed after 5 d culture, **(D)** Assessment of chondrogenic differentiation potential of PCD after 21 days’ spheroid culture, and **(E)** Estimated quantity of accumulated sGAG in the cells after 21 d.

Within 24 h of seeding, the ADMSCs moved closer to each other (Figure 6A), towards formation of clusters at day 3 (Figure 6B). Post 5 d culture, almost all the cells migrated to those clusters (Figure 6C).

In the hydrogel culture, the clusters matured into spheroids, and were evaluated for cartilage related gene (COLII, SOX 9 and aggrecan) expression using qRT-PCR analysis. In spheroid culture, expression of COLII and aggrecan (ACAN) was upregulated and SOX 9 was downregulated as compared to control at 21 d study (Figure 6D), pointing towards the potential of peptide-conjugated carbon nano-dots to stimulate cellular differentiation and tissue formation. The results of the Alcian Blue assay (Figure 6E) also suggest presence of a significantly higher quantity of sGAG in the cells cultured in the media containing peptide-conjugated carbon nano-dots (PCDs) as compared to the cells in the native carbon nano-dots (CDs) containing media, evidencing the strong chondro-inductive abilities of the synthesized PCDs.

### 3.7 *In vivo* rabbit auricular cartilage defect healing

**FIGURE 7:**
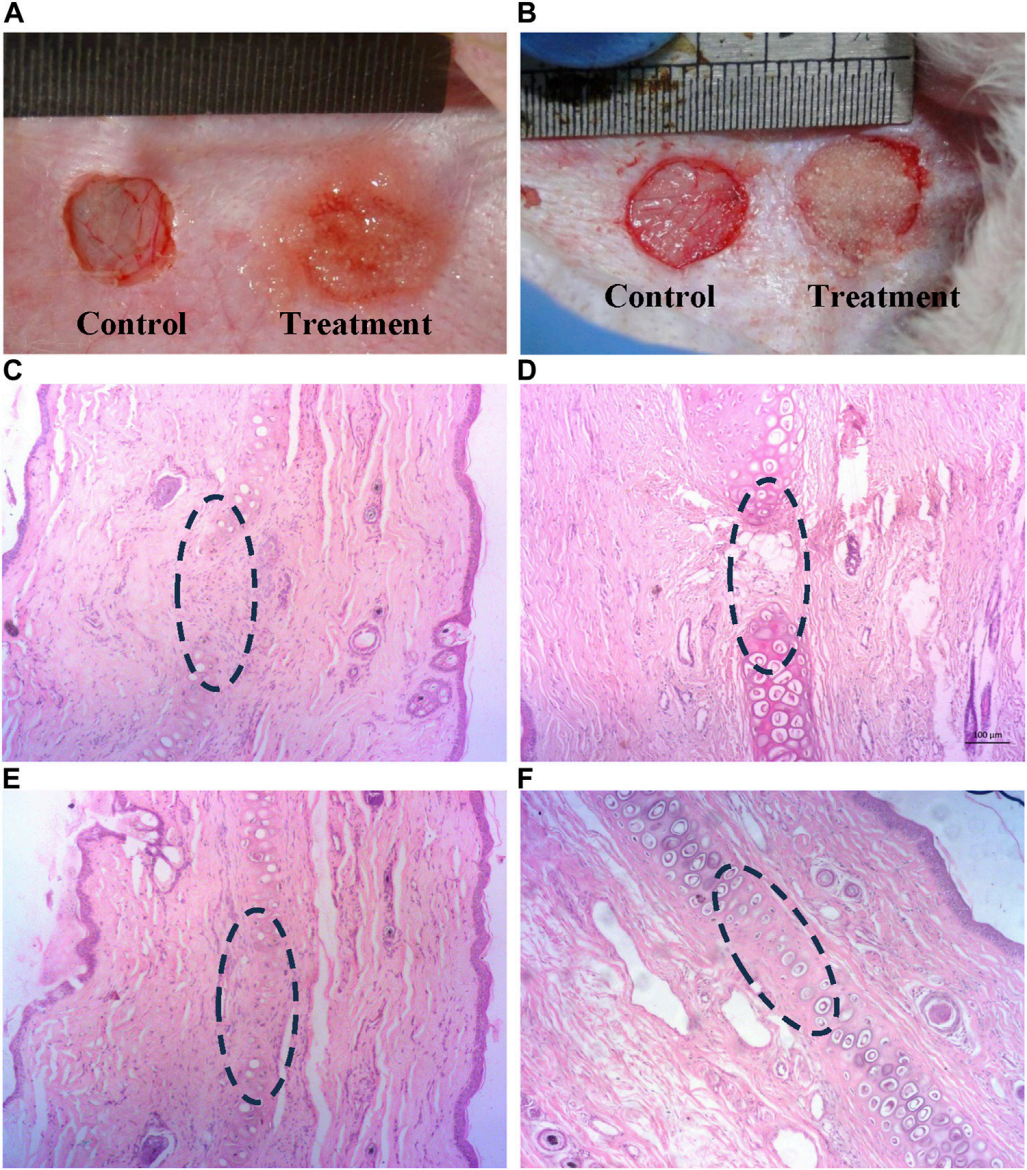
Histological analysis of *in-vivo* cartilage regeneration induced by PCD: **(A,B)** Defected auricular cartilage with one control and the other treated with PCD-hydrogel, **(C,E)** Control area showing no noticeable presence of chondrocytes at 15 and 60 d, respectively, in the defect area in the center of the images **(D,F)** Presence of chondrocytes and cartilage regeneration at the PCD-treated area at 15 and 60 d, respectively (All images were taken at ×10 magnification).

Gross observation of auricular cartilage defect: There were no apparent signs of infection or graft rejection at the defect region. Thicker skin tissue formation in PCDs group was observable as compared to the control groups 15 d post-surgery (Figure 7A). On 60 d evaluation, presence of dense cartilage tissue could be identified in defect area of PCDs group whereas thick skin remained in the control group (Figure 7B), without any cartilage tissue.

Histological analysis: H&E-stained sections of retrieved specimens at 15 and 60 d revealed that the defect region of auricular cartilage in the control area did not show any presence of chondrocyte at both 15 and 60 d post-surgery (Figures 7C, D), and the treatment area was also almost devoid of chondrocytes 15 d post-implantation of PCDs-laden hydrogel (Figure 7E), but the specimens taken 60 d post-implantation evidenced significant regeneration of cartilage tissue with strong presence of chondrocytes (Figure 7F).

**FIGURE 8:**
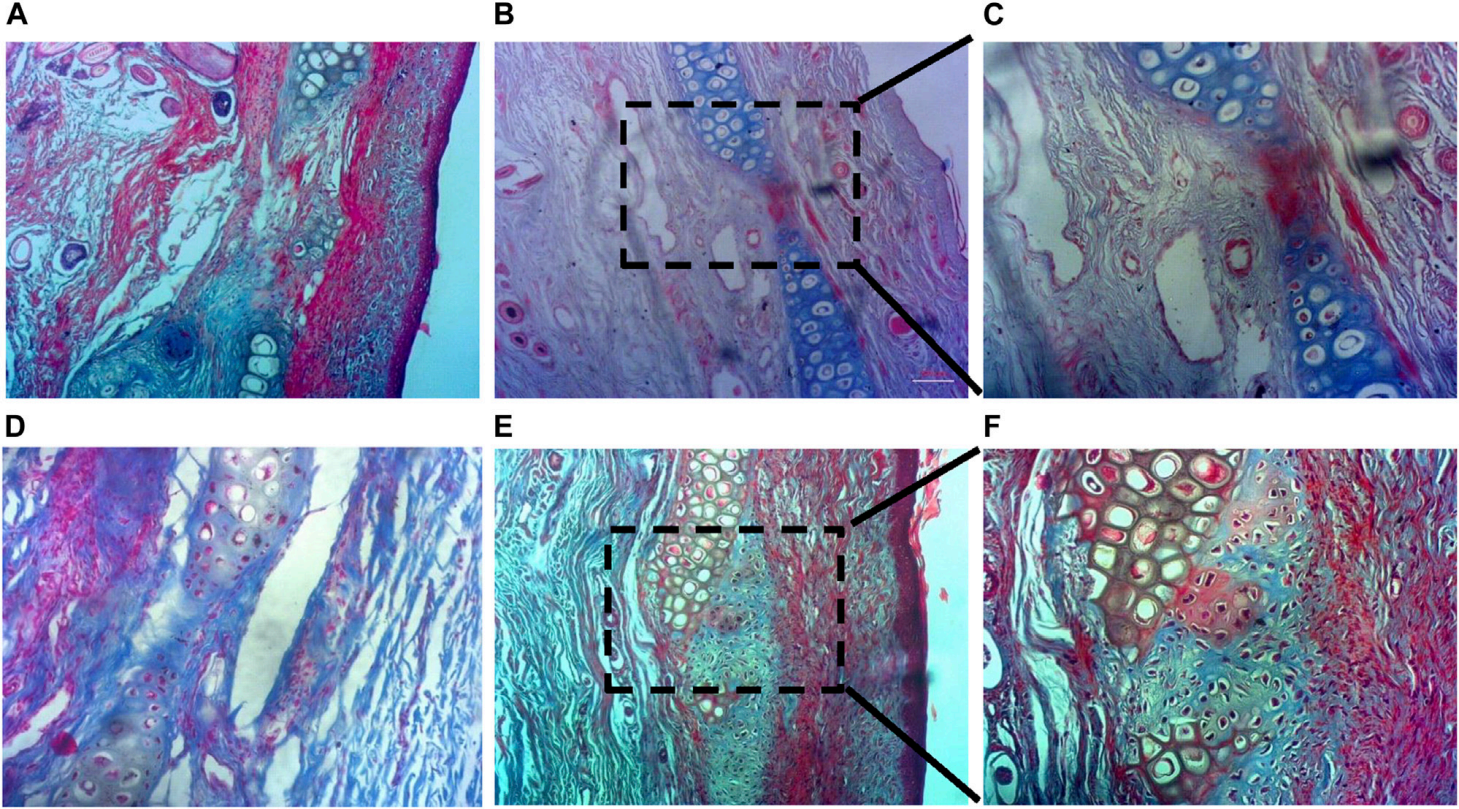
Histochemical analysis of cartilage regeneration in the defect area. **(A,D)** No detectable cartilage regeneration in the control group at both 15 d and 60 d post-implantation, **(B,C)** Reduced defect size with new cartilage formation at the treated zone within 15 d, and **(E,F)** Neo-cartilage formation with presence of formed lacunae at the treated zone 60 d post-implantation (a, b, d, and e are ×10 magnified images; c and f are ×20).

The histochemical analysis of cartilage regeneration in the defect area is presented in Figure 8. Since the Alcian blue stain binds strongly to sulfated GAGs and glycoproteins, and cartilage tissue contains the highest concentrations of GAGs than any other tissue, newly-formed cartilage appears blue in the MT-stained tissue samples. No significant tissue growth could be observed in the control group, both 15 d and 60 d post-implantation (Figures 8A, D, respectively). No capsular layer was observable in any of the tissues, indicating healthy integration of newly-formed tissues with existing ones and absence of fibrosis. 15 d study of the treated zone showed growth of neo-cartilage with a reduced defect size (Figures 8C, D), whereas dense cartilage with structurally formed lacuna was prominent in the treated defect 60 d post-implantation (Figures 8E, F).

## 4 Conclusion

In conclusion, this work presents development of nucleus-targeting zwitterionic CDs from the auricular cartilage of *Capra hircus*, and establishes the efficacy of the CDs as a vehicle for peptide delivery towards cellular modulation as well as cartilage defect regeneration. The CDs were also found to exhibit excitation-dependent photoluminescence, which can be exploited for monitoring and tracking of these CDs. Therefore, the developed CDs has multifunctional utilities including biomolecule delivery, live cell imaging and non-invasive monitoring of healing and tissue in-growth in defect area. Additionally, the peptide-conjugated CDs (PCDs) revealed strong potential to induce chondrogenic differentiation in ADMSCs, and could also facilitate cartilage regeneration in rabbit model. Thus, the CDs and the peptide-conjugated variety of them can be potential diagnostic and treatment option for cartilage defects.

## Data Availability

The raw data supporting the conclusion of this article will be made available by the authors, without undue reservation.
