# Supplementary material for: Capra cartilage-derived peptide delivery via carbon nano-dots for cartilage regeneration
Source: Front Bioeng Biotechnol. 2023 Aug 28;11:1213932. doi: 10.3389/fbioe.2023.1213932 (PMC10493328; doi:10.3389/fbioe.2023.1213932)
Supplement: Supplementary file 1 [file DataSheet1.PDF]

Table A.1. Primers used for qRT-PCR analysis

Genes	Sequence	T_m	Product Size (bp)
GAPDH-FP	*TGAGATCAAGAAGGTGGTGAAG*	62	123
GAPDH-RP	*GCATCGAAGGTAGAAGAGTGAG*		
COLIIA1-FP	*GGCCTGTCTGCTTCTTGTAA*	62	99
COLIIA1-RP	*GTCCTAGAGTGACTTGGATTGG*		
AGGRECAN-FP	*CCTCAGGGTTTCCTGACATTAG*	62	99
AGGRECAN -RP	*GCTCAGTCACGCCAGATATT*		
SOX9-*FP*	*GAATGCTTCAGCAGCCAATAAG*	62	108
SOX9-*RP*	*CAGGTGATGGTGTTAGTGAGAG*		

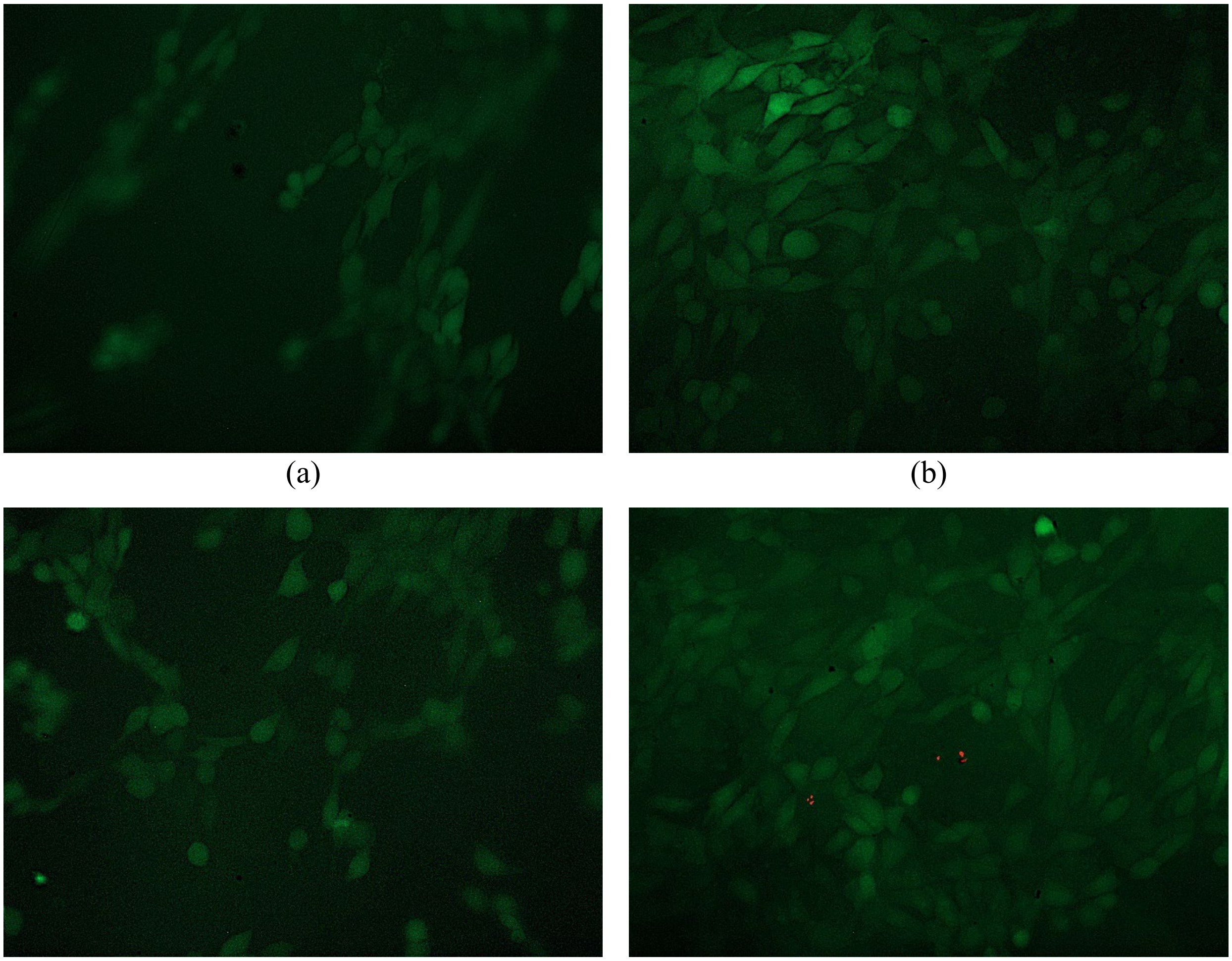

(a) (b)

(c) (d)

Fig. A.1 (a, b) Live-Dead assay of MG63 cells in CD-containing media at one and five days of culture, respectively, and (c, d) Live-Dead assay of MG63 cells in PCD-containing media at one and five days of culture, respectively
